# Nontuberculous Mycobacterial Ocular Infections: A Systematic Review of the Literature

**DOI:** 10.1155/2015/164989

**Published:** 2015-05-27

**Authors:** Wajiha J. Kheir, Huda Sheheitli, Maamoun Abdul Fattah, Rola N. Hamam

**Affiliations:** Department of Ophthalmology, Ocular Immunology and Uveitis Service, American University of Beirut, Riad El Solh, Beirut 1107 2020, Lebanon

## Abstract

Nontuberculous or atypical mycobacterial ocular infections have been increasing in prevalence over the past few decades. They are known to cause periocular, adnexal, ocular surface and intraocular infections and are often recalcitrant to medical therapy. These infections can potentially cause detrimental outcomes, in part due to a delay in diagnosis. We review 174 case reports and series on nontuberculous mycobacterial (NTM) ocular infections and discuss etiology, microbiology, risk factors, diagnosis, clinical presentation, and treatment of these infections. History of interventions, trauma, foreign bodies, implants, contact lenses, and steroids are linked to NTM ocular infections. Steroid use may prolong the duration of the infection and cause poorer visual outcomes. Early diagnosis and initiation of treatment with multiple antibiotics are necessary to achieve the best visual outcome.

## 1. Introduction

Nontuberculous mycobacteria (NTM) are defined as mycobacteria other than* Mycobacterium tuberculosis*. NTM infections are found ubiquitously in the environment in soil, dust, and water [1, 2]. Human infection is thought to be acquired from environmental exposures [1].

Nontuberculous or atypical mycobacterial ocular infections were first reported in a case of* Mycobacteria fortuitum* keratitis by Turner and Stinson in 1965 [3]. Reports of these infections increased in frequency and variety over the years, with cases reported of choroiditis [4] in 1969, orbital infections [5] in 1969, and endophthalmitis [6] in 1970, to list a few. With the advent of laser-assisted in situ keratomileusis (LASIK), nontuberculous mycobacteria began to be further implicated in cases of keratitis [7]. In more recent years, these infections have become subject to much study considering their potentially detrimental outcomes.

NTM infections are difficult to identify, with a significant delay in diagnosis or initial misdiagnosis causing a delay in treatment [8]. Their course is indolent, additionally prolonged with the use of topical corticosteroids [9], and often refractory to multiple medical therapies and surgical interventions. Timely diagnosis and proper treatment of these infections are paramount. In this paper, we systematically review 174 case reports and case series of 420 eyes from 379 patients infected with NTM. Etiology, microbiology, risk factors, diagnosis, clinical presentation, and treatment of the different types of ocular infections are discussed.

## 2. Methodology

**Figure 1:**
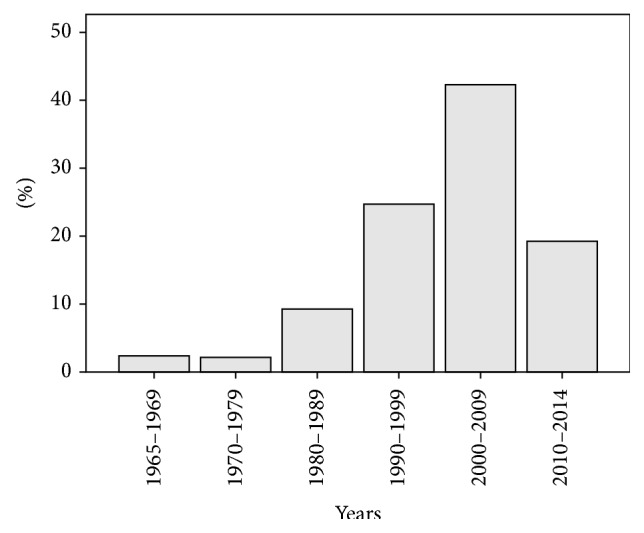
Distribution of reports of NTM infections over time.

Pubmed, Medline, and Scopus databases were accessed in November 2014 and a thorough search of the literature was conducted. The keywords* mycobacter* ^*^ *, atypical or nontuberculous or non-tuberculous, or avium or intracellulare or avium-intracellulare complex or avium complex or leprae or malmoense or marinarum or scrofulaceum or simiae or szulgae or ulcerans or xenopi or abscessus or chelonae or fortuitum or gordonae or smegmatis or ulcerans or massiliense, ophthalmic or eye or ocular or ophthalmological or eye disease* and Medical subject headings (MeSh)* nontuberculous mycobacteria* and* eye infections* were used. Non-English language case reports/series were excluded from the review. No other exclusion criteria were employed. 174 eligible case reports and series on NTM ocular infections were identified from August 1965 to September 2014. The distribution of these papers over time can be found in Figure 1. Additional three non-English reports were excluded.

From each paper, information on patient characteristics and course of infection was collected. Patient characteristics included age, gender, past medical history, past ocular history, and immune status. Information related to the course of infection was laterality, location, onset, initial and final visual acuity, clinical manifestations, type of samples taken, need for additional samples, tests determining diagnosis, delay in diagnosis, pathology, result of acid-fact bacillus (AFB) stain, type of organism, coinfection, possible source of infection, preceding interventions, associated trauma or foreign body, use of steroids, implants and contact lenses, medical and surgical treatment, prolonged course of infection, type and mode of delivery of antibiotics, duration of treatment, and outcome.

## 3. General Results

### 3.1. Location of Infection

NTM has been reported to cause periocular and adnexal infections, ocular surface infections, intraocular infections, and uveitis, as summarized in Table 1.

There exist several case reports of NTM infections involving more than one structure in the eye. Clare and Mitchell reported a case of iris root abscess and necrotizing sclerokeratitis in an immunocompetent woman with no apparent risk factors [177]. Sclerokeratitis was seen after cataract surgery in 4 eyes [40, 123], and keratitis leading to endophthalmitis was also seen in 6 eyes after cataract surgery [157, 158, 161] and 2 eyes after penetrating keratoplasty (PKP) [133, 156]. In one case of an elderly lady with dry eyes, punctal plug insertion caused secondary upper lid canaliculitis and keratitis [29]. Furthermore, endophthalmitis with a preseptal abscess was reported after cosmetic contact lens use in an eye with a failed corneal graft [164].

### 3.2. Patient Demographics

A total of 379 patients were reviewed. There was no gender predominance in NTM infections with a female to male ratio of 0.96 (*n* = 368). The average age of patients was 46 years (median 44, range 5–89) (*n* = 369). If we look at the distribution of infections across age groups, 19 patients (5.1%) were below age 20, 244 patients (66.1%) were between ages of 20 and 59, and 106 patients (28.7%) were above age of 60.

### 3.3. Microbiology

In our review, the culprit organism was found to be* M. chelonae* in 179 eyes (42.7%),* M. abscessus* in 46 eyes (11.1%), and* M. fortuitum* in 62 eyes (14.7%). These 3 species in addition to other NTM species we encountered are listed in Table 2 with their frequencies.

## 4. Results Specific to Location of Infection

A summary of the results specific to location of infection can be found in Table 3.

### 4.1. Orbital Infections

#### 4.1.1. Etiology

NTM orbital infections are quite rare; only 11 cases have been reported in the literature [5, 10–16]. Approximately half the cases were preceded by an intervention (5/11 eyes, 45.5%); orbital reconstruction and fracture repair after trauma in 4 eyes (36.4%) and blepharoplasty in 1 eye (9.1%). Orbital implants were implicated in 5/11 eyes (45.5%).

#### 4.1.2. Clinical Presentation

Patients presented with a variable course ranging from days to months. Time to presentation was on average 8 weeks (range: 3 days–44 weeks). Patients presented with an intraconal mass gradually causing restricted motility and proptosis in 2/11 eyes (18.2%). Periorbital cellulitis with secondary orbital abscesses/lesions was seen in 2 eyes (18.2%). Associated osteomyelitis of the frontal bone with bone erosion was also seen in 2 eyes (18.2%), while infections that occurred after enucleation with Teflon ball implantation led to Teflon ball implant exposure or extrusion in 2 eyes (18.2%).

#### 4.1.3. Diagnosis

Diagnosis of orbital NTM infections was achieved through culture of specimens obtained from the site of infection in 9/10 eyes (90%). DNA sequence analysis had to be done to confirm the diagnosis in 1/10 eyes (10%). The types of specimens used to make the final diagnosis were purulent material collected through simple swab in 1/7 eyes (14.2%) or drainage of abscesses in 2/7 eyes (28.5%) and orbital tissue biopsy of associated lesions in 4/7 eyes (57.1%). Pathology specimens revealed a chronic inflammatory lesion with granulomatous noncaseating features. Acid fast bacilli were revealed in the area of necrosis and were also found in lipid vacuoles in cases where there was orbital fat involvement.

Authors referred to status of delay in diagnosis of NTM infection in only 4 eyes. The reported delay was due either to not sending samples for culture initially or to the fact that no growth on culture once sample was sent or to misdiagnosis. For example, an orbital infection was confused for pseudotumor causing a 26-week delay in proper diagnosis and therefore effective treatment [11].

#### 4.1.4. Treatment

Orbital NTM infections were treated with systemic antibiotics alone in 3/11 eyes (27.3%) or in combination with surgery in 8/11 eyes (72.7%). Most infections required the use of more than two types of antibiotics (6/11 eyes, 54.6%), most commonly combinations of macrolides, fluoroquinolones, and amikacin. Of the 8 eyes that underwent surgery, 3 had excision of the infectious lesion, 1 had a simple incision and drainage, and 1 had to undergo removal of an orbital implant. In fact, out of the 4 eyes that did not respond to initial medical therapy, 3 had to undergo a surgical intervention.

#### 4.1.5. Outcome

Orbital NTM infections had a variable prognosis. Treatment led to complete resolution of the infection with no loss of vision in 7/10 eyes (70%). However, 3 eyes had a final visual acuity of 20/200 or worse, thereby rendering them legally blind. In our review, a prolonged course was determined as initial failure of medical therapy or more than one required surgical intervention. Four eyes with orbital NTM infections had a prolonged course (4/10 eyes, 40%), but the infection eventually resolved with no loss of vision in these eyes. There were no reported cases requiring enucleation, evisceration, or exenteration.

### 4.2. Eyelid/Periocular Skin Infections

#### 4.2.1. Etiology

Twenty-eight cases of eyelid and periocular skin infections have been reported [14, 17–25]. All with the exception of 1 eye had a preceding intervention (27/28 eyes, 96.4%). The most common interventions were ptosis repair and/or blepharoplasty in 22 eyes (78.6%) and dacryocystorhinostomy (DCR) with or without stent placement in 4 eyes (14.3%). There were isolated cases of infections in eyes preceded by reconstruction/fracture repair after trauma and chalazion excision. Implants were implicated in 9/28 eyes (32.1%), and these included orbital implants, fat injections, lacrimal plug or stents, and silicone rods depending on the type of preceding intervention.

The one case that was not related to an intervention was that of a young man who got* M. marinum* preseptal cellulitis of his lower and upper lids after self-manipulating a hordeolum. The authors attributed his infection to exposure from his work place, a tropical fish shop [17].

#### 4.2.2. Clinical Features

Eyelid and periocular skin NTM infections typically had a subacute presentation. However, reported symptoms ranged from immediate postoperative to month after any prior intervention (1 week–12 weeks). Presenting symptoms were mainly firm erythematous single and multiple nodular lesions along surgical wounds in 17/24 eyes (70.8%) and were associated with progressive swelling and surrounding periorbital cellulitis. Frequently, the red nodules drained purulent discharge. Elevated infectious nodules also presented without erythema or other inflammatory signs in 7/24 eyes (29.1%). There were no associated intraocular manifestations.

#### 4.2.3. Diagnosis

The diagnosis of atypical mycobacterial infection was made by culture in 23/26 eyes (88.5%) or on histopathologic examination in 3 eyes (11.5%). Histopathologic findings included chronic granulomatous inflammation and necrotizing granulomata. The specimen used to make the diagnosis was the purulent discharge collected by swab in 5/15 eyes (33.3%). Other cases required incision and drainage in 4/15 eyes (26.7%) as well as biopsy of nodular lesions in 3/15 (20%) to achieve a diagnosis. In two cases diagnosis was only achieved after intraoperative tissue, taken from debrided nodules and lesions that were excised as part of the treatment, was sent for culture and histopathology. When AFB stain was performed, it was positive in 9/10 eyes (90%).

Delay in diagnosis of eyelid and periocular skin NTM infections was encountered in 6/12 eyes (50%). Reasons for the delay included no growth or slow growth on culture and not sending samples for cultures initially.

#### 4.2.4. Treatment

Treatment of eyelid and periocular skin infections consisted of either medical therapy in 8/28 eyes (28.6%) or surgical therapy in 2/28 eyes (7.1%) or a combination of both modalities in 18/28 eyes (64.3%). Regarding medical therapy, systemic antibiotics were used in all 26 eyes treated medically, with 3 eyes additionally treated with topical antibiotics. More than half the infections required more than 2 antibiotics in the regimen (14/28 eyes, 50%). These were usually a combination of amikacin, macrolides, and fluoroquinolones. Antibiotics that were used alone were fluoroquinolones in 4/28 eyes (14.3%) and macrolides in 8/28 eyes (28.6%).

Of the infections that failed to respond to initial medical therapy (14/23 eyes, 60.9%), 11 (78.6%) had to undergo surgical treatment. In fact, excision of infectious lesions alone was sufficient to clear the infection in 2/28 eyes (7.1%). Examples of the types of surgical treatment that had to be performed are excision of lesions, incision and drainage, and debridement in 13/28 eyes (46.4%). Of the 9 infections that implicated an implant, removal of that implant was necessary for the resolution of the infection in 7 eyes.

#### 4.2.5. Outcome

Patients with eyelid NTM infections had a relatively good prognosis; infection generally resolved with no major sequel affecting the eyelid function in 22/28 eyes (78.5%). There were no reported cases of loss of eye due to NTM eyelid infection. Six cases had a prolonged course but eventually recovered with no change in vision or eyelid dysfunction. There were no reported cases leading to loss of vision or enucleation, evisceration, or exenteration.

### 4.3. Lacrimal System Infections

#### 4.3.1. Etiology

There are seventeen cases of lacrimal system infections, dacryocystitis, and canaliculitis, reported to be due to NTM [14, 18, 26–34]. Most cases (14/17 eyes, 82.3%) were preceded by one of two types of interventions, punctal plug or lacrimal tube insertions in 9/17 eyes (52.9%) and DCR with or without stent placement in 5/17 eyes (29.4%). Implants were implicated in 13/17 eyes (76.5%). Previous ocular history of epiphora and nasolacrimal duct obstruction was found in the 2 eyes that had not had prior intervention. One of these eyes belonged to a patient who was HIV-positive.

#### 4.3.2. Clinical Presentation

Presentation was subacute, with onset of symptoms ranging from 2 to 26 weeks after intervention. Patients presented with epiphora and purulent discharge from the puncta, along with swelling and erythema at the medial canthal area or at the site of the DCR incision, with or without associated nodular lesions in 12/14 eyes (85.7%). Less frequently, they had blood tinged purulent discharge in 2/14 eyes (14.2%).

#### 4.3.3. Diagnosis

Diagnosis was mainly made through swab cultures taken from draining purulent material expressed from canaliculi 8/11 (72.7%). In certain situations where the draining material was insufficient for diagnosis, biopsy of the associated nodular lesion confirmed diagnosis (2/11 eyes, 18.2%). Chronic granulomatous reaction was frequently seen in the affected material and acid fast staining revealed bacilli within the area of necrosis.

Delay in diagnosis as reported by authors was seen in 6/14 eyes (42.9%). Causes of delay included delay in sending samples for culture, no growth on initial samples taken, and misdiagnosis.

#### 4.3.4. Treatment

Like eyelid and periocular skin infections, treatment of lacrimal system infections consisted of medical therapy in 2/17 eyes (11.8%), surgical therapy in 2/17 eyes (11.8%), or a combination of both in 13/17 eyes (76.5%). Surgery alone was enough to clear the infection in 2/17 eyes (11.8%). All 8/13 eyes (61.5%) that did not respond to initial medical therapy had to undergo surgery. The most common type of surgery was removal of the implant with or without debridement in 10/17 eyes (58.8%). Other surgical therapies included excision of lesions, incision and drainage, and canaliculotomy.

The most common mode of antibiotic administration was a combination of both topical and systemic (7/17, 41.2%). Topical antibiotics alone were used in 3/17 eyes and systemic antibiotics alone were used in 2/17 eyes. More than 2 types of antibiotics were used in 8/17 eyes (47%), and these included a combination of mainly amikacin, fluoroquinolones, and macrolides. When a single antimicrobial was used, choices were amikacin in 3/17 eyes (17.6%), fluoroquinolones in 2/17 (11.8%) eyes, and macrolides in 2/17 eyes (11.8%).

#### 4.3.5. Outcome

Dacryocystitis and canaliculitis also had a good prognosis. The majority of infections (13/17 eyes, 76.5%) had complete resolution of the infection. Three cases had a prolonged course with eventual resolution. One patient was lost to follow-up. There were no reported cases leading to loss of vision or enucleation/evisceration.

### 4.4. Keratitis

#### 4.4.1. Etiology

Keratitis is the most common type of ocular NTM infection, with 290/420 eyes (69%) reported so far in the literature [3, 4, 8, 27, 35–133]. The vast majority of keratitis infections are preceded by an intervention (190/273 eyes, 69.3%), most commonly LASIK in 130/273 eyes (47.6%).

Other interventions include cataract surgery in 24/273 eyes (8.8%), penetrating keratoplasty (PKP) in 26/273 eyes (9.5%), and pterygium/pinguecula excision in 4/273 eyes (1.5%). NTM keratitis has also been seen following radial keratotomy, cataract surgery with PKP, laser epithelial keratomileusis, deep anterior lamellar keratoplasty, and endokeratoplasty.

Other possible risk factors for the development of NTM keratitis are trauma (43/264 eyes, 14.8%) and presence of a foreign body (51/211 eyes, 17.6%). The most common type of foreign body implicated was metallic in 31/51 eyes (60.8%), with wood, glass, plant debris, shale, and clay accounting for a few cases. Not all authors outlined the mechanism by which the foreign body got in the eye.

Keratitis was found to be a serious complication of contact lenses, whether soft or hard. In our review, contact lenses were used in 19/276 eyes (6.4%), including one case involving a bandage contact lens. Steroids were used in more than half of the cases of keratitis (101/176 eyes, 57.4%). As for implants, they were found in 44/254 eyes (17.3%) and were mainly intraocular lenses (22/254 eyes, 8.7%) and corneal grafts/tissues (24 eyes, 9.4%).

Patients with keratitis and no other obvious risk factors were found to have certain medical problems including miliary tuberculosis, rheumatoid arthritis, bullous pemphigoid, and a history of malignancy. Relevant ocular history was determined to be ocular surface disease in 2 eyes and exposure keratopathy in 1 eye.

#### 4.4.2. Clinical Presentation

With NTM keratitis, time to presentation varied from 1 day to 1 year, with an average of 5.6 weeks (*n* = 158). On examination, patients typically exhibited a “cracked windshield” appearance of the cornea around the edge of the central area of the infiltrate. Infiltrates at times had irregular margins or satellite lesions, mimicking fungal keratitis [38, 39, 44, 64, 80, 81, 92]. Dendritic epithelial defects with minimal stromal infiltration were also seen in NTM infections, prompting authors to falsely diagnose herpes keratitis [50, 88].

#### 4.4.3. Diagnosis

The diagnosis of NTM keratitis was done mostly through culture of samples from the eye. Occasionally, polymerase chain reaction (PCR), PCR probes/hybridization, PCR restriction endonuclease analysis, and gene sequencing were needed to establish or confirm the diagnosis. AFB stain tested positive in samples from 101/115 eyes (87.8%). Regarding samples collected, isolation of the causative organism in NTM keratitis often required only a superficial corneal scraping (127/198 eyes, 64.1%). When scrapings did not reveal the organism, corneal biopsy was needed to reach a diagnosis (19/198 eyes, 9.5%). When NTM keratitis occurred after LASIK, infiltrates appeared within the lamellar flap or at the flap interface. Making a swift diagnosis required the lifting of the flap to obtain scrapings for microbiological evaluation in 27/198 eyes (13.6%). In eyes that necessitated corneal transplant, the corneal button was often used to determine diagnosis (11/198 eyes, 5.5%). In isolated cases, surgical instruments and a lens care system were used when more traditional methods failed to offer a causative organism.

Delay in making the diagnosis was reported in 61/110 eyes (55.5%). Reasons provided for this delay were misidentification of the causative organism, delay in taking cultures, no growth or slow growth of the organism, and misdiagnosis. Organisms misidentified as the causative agent were* Nocardia* species [35, 36, 70, 71, 128] and* Corynebacterium* species [74]. Relevant misdiagnoses made were herpes keratitis [46, 50, 53, 55, 66] and fungal keratitis [39, 46, 48, 54, 94]. The duration of delay ranged from 1 week to 30 weeks, with an average of 8 weeks (*n* = 24).

#### 4.4.4. Treatment

Most cases of NTM keratitis were treated with medical therapy alone in 127/283 eyes (44.9%) or a combination of medical and surgical therapy in 156/283 eyes (55.1%). Surgical treatment was required in 156/283 eyes (55.1%). Of the infections that had an initial lack of response to medical therapy (141/193 eyes, 73.1%), 111/140 eyes (79.3%) had to undergo a surgical intervention. The most common types of surgeries were removal the corneal flap in 49/283 eyes (17.3%), PKP in 40/283 eyes (14.1%), extirpative keratectomy in 15/283 eyes (5.3%), and removal of implant, whether a corneal graft or an IOL, in 9/283 eyes (3.6%). In fact, removal of the flap for resolution of infection was needed in 49/61 eyes after LASIK (80.3%).

Results related to medical therapy were centered on treatment of infections that led to eventual resolution without severe loss of vision (final visual acuity better than 20/200). The mode of delivery and antibiotics used are summarized in Table 4. The most common modes of delivery of antibiotics were topical in 108/203 eyes (53.2%) and a combination of both topical and systemic in 85/203 eyes (41.9%). More than two antibiotics had to be used in 112/203 eyes (55.2%), with the majority of the combinations including amikacin (80/203, 39.4%). In fact, amikacin constituted sole therapy in 56/203 eyes (27.6%). Other commonly used antibiotics were fluoroquinolones and macrolides, alone or in combination with other antibiotics.

#### 4.4.5. Outcome

The majority of cases of NTM keratitis resolved without severe loss of vision (190/235 eyes, 80.9%). Among these, 48 (25.8%) had a prolonged course that necessitated either multiple medical therapies or more than 1 surgical intervention before resolution was reached. With respect to final visual acuity, more than half of the cases had a good outcome of 20/40 or better (112/204 eyes, 54.9%). Nonetheless, NTM keratitis was a potentially debilitating infection, with 40/204 eyes (19.6%) ending up with loss of vision or legal blindness. More so, 3 cases ended up with loss of the eye (3/235 eyes, 1.3%). Patients who underwent a surgical intervention were more likely to end up with visual impairment (RR = 2.7, *P* value 0.001).

### 4.5. Scleritis

#### 4.5.1. Etiology

There are eighteen cases of NTM scleritis reported in the literature [134–143]. Almost all cases were directly preceded by an intervention (17/18 eyes, 94.4%). These included scleral buckling procedure in 14/18 eyes (77.8%) and isolated cases of a pterygium excision, an intravitreal injection, and a pars plana vitrectomy (PPV). The one case of NTM scleritis not following a procedure occurred in an immunocompromised man with severe medullar hypoplasia on interleukin-2 (IL-2) treatment. He had a disseminated* M. chelonae* infection with spondylodiscitis and spinal epidural abscess in addition to the scleritis [137].

#### 4.5.2. Clinical Presentation

In eyes that had undergone scleral buckling, NTM infections occurred weeks to months after the surgery (1.5 weeks–40 weeks). Manifestations included nonspecific symptoms of chronic redness, pain, and discharge. Infection was shown to lead to marked scleral thinning along with scleral buckle erosion and exposure, along with scleral abscess/subconjunctival nodules with mucopurulent discharge mainly late in the disease course. Scleral abscesses were seen after pterygium excision, and focal lesions were seen around the sutures at the scleral ports after vitrectomy. The immunocompromised patient presented with a nodular necrotizing scleritis. Although only conjunctival inflammation was seen after intravitreal injection, a hypoechoic excavation of the sclera at the site of the injection was evident on anterior segment ultrasound.

#### 4.5.3. Diagnosis

Diagnosis of NTM scleritis was made through samples obtained from scleral biopsies of abscesses and nodules sent for culture. In 1 case, the etiological agent was confirmed by 116S rRNA sequencing [134]. Explanted scleral buckles were also used to isolate NTM. Conjunctival biopsy specimens from vitrectomy port sites revealed the diagnosis after PPV. None of the authors reporting on time to diagnosis found a significant delay (6 eyes).

#### 4.5.4. Treatment

Eyes with NTM scleritis were treated with a combination of medical and surgical therapy in 15/17 eyes (88.2%) and medically alone in 2/17 eyes (11.8%). All 14 eyes associated with scleral buckles required surgery, 13/17 eyes had explantation of the buckle with debridement of necrotic tissue. One eye needed debridement after the removal of an exposed scleral buckle [141]. Other types of surgical interventions were debridement procedures with or without the use of scleral patch grafts for scleral thinning.

Medical therapy consisted of topical antibiotics (16/17 eyes, 94.1%), in combination with systemic antibiotics (5/17 eyes, 29.4%), periocular antibiotics (1/17 eyes, 5.8%), or both (4/17 eyes, 23.5%). Most eyes (12/17 eyes, 70.6%) were treated with 2 or more types of antibiotics. Amikacin and other aminoglycosides such as gentamicin and kanamycin were the most common antibiotics used (12/17 eyes, 70.6%), and these were usually combined with macrolides and/or fluoroquinolones.

#### 4.5.5. Outcome

Although NTM scleritis resolved on proper treatment in the majority of the cases (16/17 eyes, 94.1%), the visual outcome was poor; 10/14 eyes (71.4%) had a final visual outcome of 20/200 or worse. Of the cases that resolved, 2/16 eyes (12.5%) had a prolonged course requiring a change in the medical treatment regimen in both and an additional surgery for debridement in one. Only 1 case (1/17 eyes, 5.9%) ended up with loss of the eye.

### 4.6. Conjunctivitis

There were only 3 cases of isolated NTM conjunctival involvement reported in the literature [144–146]. One occurred in an AIDS patient with bacillary angiomatosis of the palpebral conjunctiva with coinfection by NTM and cytomegalovirus. He presented with a large mass protruding from his eye, which underwent debulking. Cultures of intraoperative specimens revealed the diagnosis. On pathology, acid fast bacilli (AFB) were concentrated in areas of microabscesses. He was treated with topical erythromycin and systemic azithromycin, ethambutol, isoniazid, rifampicin, pyrazinamide, and acyclovir. The mass resolved with no effect on visual acuity [144].

A second case occurred in a healthy middle-aged woman who raised parrots and wore soft contact lenses but did not have any risk factors otherwise. She presented with a large, fleshy, elevated conjunctival mass that was treated with excision and topical ciprofloxacin and amikacin. After conventional staining techniques failed, diagnosis of* M. fortuitum* was established by PCR. Pathology revealed suppurative granulomatous inflammation. A recurrence was successfully treated with repeat excision and oral clarithromycin and moxifloxacin [145].

The last case occurred in an elderly man who developed nodular bulbar conjunctivitis 6 weeks after cataract surgery with IOL placement. He had no apparent risk factors other than steroid treatment postoperatively. An incisional biopsy, revealing suppurative granulomas with AFB, suggested mycobacterial infection. A swab culture later confirmed infection with* M. abscessus*. After failure of oral antimicrobial therapy, the patient was successfully treated with topical ciprofloxacin and lubrication [146].

### 4.7. Endophthalmitis

#### 4.7.1. Etiology

There were 44 cases of endophthalmitis reported in the literature [6, 74, 92, 106, 116, 133, 138, 147–172]. Most were preceded by an intervention (28/37 eyes, 75.7%), mainly cataract surgery with IOL insertion (18/37 eyes, 48.6%). Other predisposing procedures were penetrating keratoplasty, intravitreal injection, scleral buckling, filtering surgery, and Descemet's stripping automated endothelial keratoplasty or DSAEK (1 eye, 2.7%). Implants were implicated in 26/37 eyes (70.3%) and included IOLs, glaucoma filtering tubes, corneal grafts/tissues, and a scleral buckle.

Possible etiological factors for eyes that did not have a direct intervention were identified. NTM endophthalmitis occurred in 6/10 eyes (60%) of immunocompromised patients, as well as in 3/10 eyes (30%) of patients with disseminated NTM infections. Other factors were steroid use in 4/10 eyes (40%), contact lens use in 1/10 eyes (10%), and an old glaucoma filtering tube implant in 1/10 eyes (10%).

#### 4.7.2. Clinical Presentation

For eyes that were preceded by a procedure, patients presented within days and up to 35 weeks after intervention, with an average of 11.5 weeks (*n* = 13). On examination, anterior chamber reaction with hypopyon was seen in 19/32 eyes (59.3%). Granulomatous keratic precipitates were also observed in 2/32 eyes (6.3%). 10/32 eyes (31.2%) had an associated vitreous inflammatory reaction. Postcataract NTM endophthalmitis revealed whitish fluffy plaque like material on the intraocular lens in 1 eye. Corneal infiltrates and/or abscesses formed in the needle-knife tract of the cataract wound in 3/32 eyes (9.2%).

#### 4.7.3. Diagnosis

In patients with NTM endophthalmitis, diagnosis was achieved mostly through culture of aqueous samples from anterior chamber tap in 11/36 eyes (30.5%) and vitreous samples from tap in 13/36 eyes (36.1%) and vitrectomy in 6/36 eyes (16.7%). Samples that underwent AFB staining tested positive in 19/20 eyes (95%). In six eyes with endophthalmitis associated with keratitis, corneal specimens were sufficient to establish the diagnosis.

On histopathology of the eyes requiring enucleation or evisceration, eye contents revealed extensive infiltration of the anterior chamber and vitreous cavity with a dense granulomatous reaction.

Delay in the diagnosis of NTM endophthalmitis was reported in 12/25 eyes (48%). Reasons identified for this delay were misidentification of the causative organism, no or slow growth on culture, and misdiagnosis. For example, chronic endophthalmitis has been mistaken for granulomatous iridocyclitis [151]. The duration of delay varied from 3 to 9 weeks and was 5.6 weeks on average (*n* = 5).

#### 4.7.4. Treatment

All eyes with endophthalmitis were treated medically, and 32/37 eyes (86.5%) were also treated with surgery. In fact, out of the 24 eyes that did not respond to initial medical therapy, 22 (91.7%) required surgical intervention. Types of surgeries were PPV in 13/37 eyes (35.1%), removal of ocular implant in 8/37 eyes (21.6%), enucleation of 5/37 eyes (153.5%), and evisceration of 3/37 eyes (8.1%).

The most common route of antibiotic administration was intraocular combined with topical/systemic/periocular antibiotics in 14/38 eyes (36.8%), topical in 8/38 eyes (21.1%), topical combined with systemic/periocular in 7/38 eyes (18.4%), and intraocular in 5/38 eyes (13.2%). Regarding the types of antibiotics, amikacin alone (5/38 eyes, 13.2%) or in combination with other antibiotics (19/38 eyes, 50%) was the most used. Combinations of antibiotics included fluoroquinolones in 7/38 eyes (18.4%) and macrolides in 11/38 eyes (28.9%). At least 2 types of antibiotics were used in 29/38 eyes (76.3%).

#### 4.7.5. Outcome

The prognosis of NTM endophthalmitis is poor. Loss of eye occurred in 12/36 eyes (33.3%) and loss of vision in 12/36 eyes (33.3%). Resolution of the infection took place in 12/36 eyes (33.3%) and, of these, 5 had a prolonged course. With respect to final visual acuity, only 5/22 eyes (22.7%) had a visual acuity of 20/40 and better. One patient with AIDS and a disseminated* M. avium* infection passed away [150]. We found no significant correlation between PPV and visual outcome.

### 4.8. Uveitis

#### 4.8.1. Etiology

There are 9 reported cases of uveitis caused by NTM in the literature: choroiditis in 6 eyes [4, 173–176], iridocyclitis in 1 eye [177], and granulomatous panuveitis in 2 eyes [178]. Predisposing factors included an intervention of cataract with PPV in 1/9 eyes (10%) and treatment with steroids in 3/7 eyes (42.9%). Uveitis due to NTM occurred in 5/9 eyes (55.6%) of immunocompromised patients, all of whom had HIV/AIDS. 3/9 eyes (33.3%) were patients with a disseminated NTM infection or localized infection elsewhere in the body. Regarding ocular history, 3/4 eyes (75%) had history of previous CMV retinitis.

#### 4.8.2. Clinical Presentation

Cases with systemic NTM infections led to the development of choroidal and other intraocular lesions in 3/9 eyes (33.3%). Choroiditis presented as multiple yellowish, round, subretinal pigment epithelial (sub-RPE) lesions. Along with the multifocal choroiditis, eyes exhibited associated panuveitis with anterior chamber reaction, iris nodules, and vitritis in 2/9 eyes (22.2%). In a patient with AIDS, the presentation was subclinical in nature prior to initiation of HAART therapy, with minimal evidence of inflammation. After initiation of therapy, massive granulomatous inflammation and panuveitis resulted bilaterally [176].

In one case of hemorrhagic anterior uveitis, a subconjunctival mass extending to the cornea turned out to be a nodular iris root abscess extending from the anterior chamber to the iris and ciliary body on ultrasound [177].

#### 4.8.3. Diagnosis

Culture of ocular samples from eyes with uveitis recovered NTM in 3/8 eyes (37.5%). Molecular techniques such as PCR confirmed the diagnosis in 3/8 eyes (37.5%). NTM were not retrieved in 2/8 eyes (37.5%), and the diagnosis was made because the patients had systemic NTM infections. Microbial analysis was carried out on vitreous samples in 5/6 eyes (83.3%) and on a corneal biopsy in 1 eye with the iris root abscess. The result of the AFB stain was mentioned in 4 eyes, all of which had a positive result. On histopathology of enucleated eyes, choroidal lesions had a suppurative center surrounded by granulomatous inflammation.

Delay in diagnosis was encountered in 5/6 eyes (83.3%). Reasons reported for the delay were misdiagnosis, misidentification of the organism, and delay in taking cultures. In one eye,* Nocardia* species was initially thought to be the causative organism [177]. In another, choroiditis was mistaken for ocular lymphoma [175].

#### 4.8.4. Treatment

NTM uveitis was treated medically, with 3/8 eyes (37.5%) requiring surgical treatment. Types of surgeries were PPV, evisceration, and enucleation. Lack of initial resolution on medical therapy occurred in 3/7 eyes (42.9%), all of which had to undergo surgical intervention. Regarding medical therapy, mode of administration of antibiotics was systemic in 3/9 eyes (33.3%), topical and systemic in 3/9 eyes (33.3%), topical, systemic and intraocular in 2/9 eyes (22.2%), and systemic, intraocular, and periocular in 1/9 eyes (11.1%). All eyes were treated with 2 or more types of antibiotics. A common treatment regimen consisted of antituberculous medications in 4/9 eyes (44.4%), while others were combinations of macrolides, amikacin, and fluoroquinolones in 5 eyes (55.6%).

#### 4.8.5. Outcome

Treatment of NTM uveitis resulted in resolution in 4/9 eyes (44.4%), with a prolonged course in 1 eye. Loss of eye occurred in 3 cases and patient passed away in 2 cases. One eye had stable choroidal lesions at the time of reporting. Final visual acuity was reported as 20/40 or better in 2/4 eyes (50%) and 20/50 in 2/4 eyes.

## 5. Discussion

Despite the increasing reports on NTM ocular infections, they remain relatively uncommon. The most frequently reported type of infection caused by NTM is keratitis and accounts for 69% of all eyes reviewed [3, 4, 8, 27, 35–133]. On the other hand, Fong et al. analyzed the clinical and mycobacterial characteristics of 476 cases of microbial keratitis at the National Taiwan University Hospital over a 10-year period (January 1992 to December 2001). NTM accounted for only 7.9% of culture positive isolates [179].

Incidence of NTM keratitis may have increased with the advent of LASIK. The American Society of Cataract and Refractive Surgery (ASCRS) Cornea Clinical Committee published data on the incidence, microbiology, treatment, and outcomes collected from a post-LASIK infectious keratitis survey. NTM accounted for 33/69 (48%), the majority of culture positive cases [180]. A study from Spain on 204,589 LASIK procedures conducted by Llovet et al. did not find any mycobacterial infections among the 72 eyes that developed infectious keratitis. The authors postulated, however, that the 33 samples with negative cultures had a late presentation and may likely be due to atypical organisms like NTM [181].

While most cases of NTM keratitis were sporadic, few have been due to outbreaks. Winthrop et al. investigated NTM keratitis outbreaks after LASIK from August 2000 to June 2001 and reported on 3 clusters of infections [182]. One outbreak was linked to the use of corneal masks from contact lens fragments [43], while another was linked to* M. szulgai* contaminated ice used to chill syringes utilized in flap lavage [54, 183]. Another cluster of 5 patients presenting with* M. chelonae* keratitis after PKP has been reported. Although the source of the outbreak was not traced, all donor corneas were harvested from the same collection center [113].

In our review, we identified several potential risk factors for ocular NTM infections, namely, any history of interventions, trauma, foreign body, implant, contact lens wear, and steroid use. When these factors were excluded from the analysis, observations on relevant past medical and ocular history were made in order to determine any added risks. Five patients had HIV/AIDS [18, 144, 154, 176, 178], and four patients had an NTM infection, either disseminated or at another site in the body [147, 150, 167, 175]. Individual cases of miliary tuberculosis [6], rheumatoid arthritis [38], bullous pemphigoid [66], and history of cancer [34] were also reported. Regarding relevant ocular history, two patients had nasolacrimal duct obstruction [18, 34], two had ocular surface disease [38, 66], and one had exposure keratopathy [66].

Of the diverse clinical presentations of ocular infections, certain ones would be suggestive of an NTM infection. An indolent infection with a delayed onset after intervention should raise the suspicion for NTM as the causative organisms. Clinical clues were especially evident in NTM keratitis; a “cracked windshield” appearance around the edge of the central area of infiltrates is practically pathognomonic. Other features such as irregular margins or satellite lesions mimicking fungal keratitis or dendritic epithelial defects mimicking herpes keratitis were also reported. If these are encountered in patients not responding to the corresponding antifungal or antiherpetic therapies, then NTM should be on the differential and therefore be investigated.

NTM have been divided into 4 Runyon groups based on their pigment and photoreactivity (Group I, photochromogens; Group II, scotochromogens; Groups III and IV, nonchromogens). Groups I, II, and III NTM are slow growing mycobacteria with a growth rate of 2 to 4 weeks. Group IV NTM are rapidly growing with a growth rate of 7–10 days [1, 2, 40, 95]. The rapidly growing* M. chelonae*,* M. abscessus*, and* M. fortuitum* were the organisms isolated from most ocular infections we reviewed. This is consistent with previous reports about the prevalence of ocular infections with these clinically important species [184, 185].

NTM species have different antimicrobial susceptibilities which makes species identification of NTM clinically important [1]. Identification of NTM is conventionally based primarily upon growth rate, pigmentation, colonial morphology, and results of biochemical tests such as arylsulfatase, catalase, and niacin tests. More recently, molecular and genetic techniques such as polymerase chain reaction (PCR), gene sequencing, and molecular probes have been used to characterize these organisms [2].

Regarding the staining of different specimens for AFB, the fluorochrome technique, the Ziehl-Neelsen, and the Kinyoun stain are used. NTM may appear pleomorphic, showing as long filaments or coccoid forms, with uniform staining properties. It is important to note that nonmycobacterial organisms, including* Rhodococcus* species,* Nocardia* species, and* Legionella micdadei*, as well as* Microsporidium* spores and the cysts of* Cryptosporidium, Isospora, *and* Cyclospora* may show various degrees of acid fastness. In this review, we came across 8 isolates of NTM ocular infections that were initially misidentified as* Nocardia* species [35, 36, 70, 71, 128, 177]. However, considering the high rate of positive AFB stains we found, reaching up to 95% in cases of NTM endophthalmitis, we recommend AFB stain as an initial diagnostic test pending microbial culture results.

When sending isolates for culture with the suspicion of NTM, both solid and broth (liquid) media should be included. The recommended broth system is the mycobacteria growth indicator tube (MGIT), and the recommended solid media include Löwenstein-Jensen and Middlebrook (7H10 and 7H11) media [1].

Isolates grown on culture media can be speciated by genotypic and molecular techniques as they allow for a rapid identification of NTM. In our review it is evident that there is increasing utilization of PCR and PCR probes/hybridization to accurately and rapidly identify specific NTM species [54, 61, 87, 121, 130, 144, 145, 151, 154–156, 178]. Another genotypic method that was frequently employed is the PCR restriction endonuclease analysis [35, 45, 165]. DNA sequencing and identification of the signature sequences of the 16S rRNA gene was also used for the initial diagnosis and confirmation of culture results [11, 81, 103, 136, 168, 169, 177]. Furthermore, the restriction fragment length polymorphism (RFLP) technique using the IS*6110* repetitive sequence as a probe [1, 2] was utilized by Palani et al. [161].

NTM can lead to a wide range of ocular infections requiring various sampling techniques for obtaining isolates for organism identification. Getting the appropriate sample for microbial analysis is critical. When swab cultures of infection sites are negative, incision and drainage or biopsy of lesions should be undertaken. In keratitis after LASIK, lifting the flap for scrapings of infiltrates at the interface is often necessary.

With all that is known about sampling and tests needed for diagnosis, it is important to determine how often delay in diagnosis was encountered in reviewed cases as well as the reasons for that delay. A delay, ranging from 1 week to 30 weeks, was seen in approximately half the cases of eyelid and periocular infections, lacrimal infections, keratitis, and endophthalmitis.

Reasons determined to play a role in the delay were no growth or slow growth of NTM, misidentification of organism, misdiagnosis, and delay in sending samples for microbiological evaluation. Some eyes had more than one reason for the delay. Examples of organisms wrongly determined to have caused the infection are* Nocardia* species [35, 36, 70, 71, 128, 177] as well as* Corynebacterium* species [74]. Some examples of the incorrect diagnoses made were herpes keratitis [46, 50, 53, 55, 66] and fungal keratitis [39, 46, 48, 54, 94]. An orbital infection was confused for pseudotumor [11]. As for intraocular infections, chronic endophthalmitis was mistaken for granulomatous iridocyclitis [151], and choroiditis was mistaken for ocular lymphoma [175].

Considering all the different causes of delay, we recommend swiftly obtaining samples for microbial analysis when infection is on the differential. Any lack of growth or slow growth encountered should prompt resorting to molecular techniques for diagnosis. Clinicians should also keep in mind the different diagnoses that NTM infections are often mistaken for while evaluating their cases.

Many studies have investigated the proper medical treatment regimen of NTM, and recently more of these have been concerned with ocular infections. Girgis et al., the largest retrospective study on ocular NTM infections to date (143 eyes), found that most isolates from NTM infected eyes were sensitive to clarithromycin (93.2%) and amikacin (81.3%), followed by linezolid (36.4%), gatifloxacin (30.9%), moxifloxacin (21.4%), and ciprofloxacin (10.3%) [184]. Another retrospective study by Brown-Elliott et al. found* M. abscessus* to be most susceptible to amikacin and clarithromycin,* M. chelonae* to amikacin, clarithromycin, and tobramycin, and* M. fortuitum* to amikacin and imipenem [186].

In vivo rabbit studies have been conducted to compare effectiveness of treatment with different fluoroquinolones; gatifloxacin was found to be synergistic with clarithromycin and amikacin [187] and found to be the most potent among fluoroquinolones [188]. In fact, the combination of amikacin and clarithromycin was not effective unless combined with gatifloxacin. In a review by Abschire et al., however,* M. chelonae* was found to be more susceptible to moxifloxacin than gatifloxacin, keeping in mind that* M. chelonae* is responsible for most NTM ocular infections [189].

Dolz-Marco et al. reported a case of* M. chelonae* resistant to amikacin, clarithromycin, and moxifloxacin, which led them to resort to linezolid to clear the infection [49]. With the threat of resistance looming demonstrated by Dolz-Marco et al., an effective treatment strategy ought to be devised. Hose et al. found no effectiveness in the combination of clarithromycin and amikacin as compared to a basic salt solution in vivo, but a combination of gatifloxacin, clarithromycin, and amikacin was found to be effective, as was treating with gatifloxacin alone [187]. Monotherapy does, however, increase the risk of resistance. We recommend treating ocular NTM infections with a combination of amikacin, a fluoroquinolone, and a macrolide pending antimicrobial susceptibilities.

Regarding surgical therapeutic interventions, we found that eyes that experienced a lack of initial response to medical therapy were 10 times more likely to undergo a surgery (*P* < 0.05). NTM infections are potentially detrimental, so any surgery deemed necessary by clinicians to clear the infection ought not be delayed.

In fact, surgery should be a prime consideration whenever an ocular implant is involved. Infections occuring after an implant was placed in the eye were more likely to necessitate surgery (OR = 3.5, *P* value < 0.001). Several therapeutic surgical interventions involved removing implants from infected eyes. Eyes with implants were almost 6 times more likely to end up with loss of vision (*P* value < 0.05), in contrast to eyes with a history of a foreign body, which were less likely to develop loss of vision (OR = 0.3, *P* value = 0.03).

Steroid use has been linked to the initial failure of medical therapy of keratitis [190]. In a rabbit model of* M. fortuitum* keratitis, eyes treated with topical corticosteroids had larger infiltrates and lesions [9]. We found that eyes which received steroids prior to diagnosis of the infection being made were almost three times more likely to have lack of initial resolution to medical therapy (OR = 2.8, *P* value = 0.001), more likely to develop a prolonged course of infection (OR = 2.7, *P* value = 0.001), and less likely to reach resolution (OR = 0.5, *P* value = 0.006). Because steroid use may lead to a prolonged course with a potentially worse outcome, we recommend its discontinuation whenever NTM infection is suspected.

Statistical analysis relating certain locations of the ocular infection with the outcome was performed. Intraocular infections were less likely to resolve (OR = 0.2, *P* value < 0.001), with a higher likelihood of undergoing a therapeutic surgical intervention (OR = 2.7, *P* value = 0.012). They were also more likely to result in loss of vision (OR = 5.3, *P* value < 0.001) and extremely more likely to result in loss of the eye (OR = 34.4, *P* value < 0.001). Predictably, ocular surface infections were less likely to result in loss of vision (OR = 0.2, *P* value < 0.001) or in loss of the eye (OR = 0.1, *P* value < 0.001). They were also less likely to require more than one therapeutic surgical intervention (OR = 0.4, *P* value < 0.001). Intraocular NTM infections, therefore, should be treated aggressively due to significant potential morbidity.

## 6. Conclusion

Nontuberculous mycobacterial infections of the eye are uncommon but are potentially detrimental. NTM can cause periocular infections, adnexal infections, ocular surface infections, intraocular infections, and uveitis, with ocular surface infections, specifically keratitis, making up the majority of cases. NTM keratitis is especially noted after LASIK procedures. The most common species causing ocular NTM infections are* M. chelonae*,* M. abscessus*, and* M. fortuitum*. Ocular NTM infections are frequently indolent and unresponsive to initial medical therapy, especially when preceded by an intervention. NTM infections are also encountered after trauma, foreign bodies, implants, and contact lens wear. Immunocompromised patients are more likely to develop intraocular NTM infections, which are associated with a greater risk of loss of vision or even loss of the eye.

Considering the potential detrimental outcomes associated with these infections, clinicians should have a high index of suspicion of NTM when faced with a challenging case. If suspecting NTM, diagnosis is made by taking appropriate samples to be sent for microbiological evaluation which includes acid fast staining and culture in liquid broth mycobacteria growth indicator tube (MGIT), or on Löwenstein-Jensen and Middlebrook (7H10 and 7H11) solid media. Molecular and genetic techniques can also be used to hasten species identification. In treatment, we recommend a combination of antibiotics based on culture sensitivities or NTM species found in order to decrease the likelihood of developing resistance. Steroid use should be avoided in suspected cases as it is associated with prolonged infections and worse visual outcomes. Therapeutic surgical intervention may be needed in order to control the infection. Timely diagnosis and initiation of therapy are key factors in achieving resolution of the infection as well as a good visual outcome.

## Figures and Tables

**Table 1:** Distribution of the types of ocular NTM infections.

Type of infection	Number of eyes (*n* = 420)
Periocular and adnexal infections	
Orbital [5, 10–16]	11 (2.6%)
Eyelid and periocular skin [14, 17–25]	28 (6.7%)
Lacrimal system [14, 18, 26–34]	17 (4.0%)
Total	**56 (13.3%)**
Ocular surface infections	
Keratitis [3, 4, 8, 27, 35–133]	290 (69.0%)
Scleritis [134–143]	18 (4.3%)
Conjunctivitis [144–146]	3 (0.7%)
Total	**311 (74.0%)**
Intraocular infections and uveitis	
Endophthalmitis [6, 74, 92, 106, 116, 133, 138, 147–172]	44 (10.4%)
Choroiditis [4, 173–176]	6 (1.5%)
Iridocyclitis [177]	1 (0.2%)
Panuveitis [178]	2 (0.5%)
Total	**53 (12.6%)**

**Table 2:** NTM species in ocular infections.

Mycobacterial species	Number of Eyes *n* = 420
*M. chelonae *	179 (42.6%)
*M. fortuitum *	62 (14.8%)
*M. abscessus *	46 (11.0%)
*M. avium complex *	8 (1.9%)
*M. szulgai *	8 (1.9%)
*M. avium *	4 (1.0%)
*M. gordonae *	4 (1.0%)
*M. immunogenum *	4 (1.0%)
*M. haemofilis *	3 (0.7%)
*M. kansasii *	3 (0.7%)
*M. massiliense *	3 (0.7%)
*M. chelonae-fortuitum *complex	2 (0.5%)
*M. mucogenicum *	2 (0.5%)
*M. aurum *	1 (0.2%)
*M. flavescens *	1 (0.2%)
*M. goodii *	1 (0.2%)
*M. houstonense *	1 (0.2%)
*M. intracellulare *	1 (0.2%)
*M. marinarum *	1 (0.2%)
*M. phlei *	1 (0.2%)
*M. smegmatis *	1 (0.2%)
**Unknown NTM species**	84 (20.0%)

**Table 3:** Summary of the results specific to location of infection.

Location of NTM infections	Total cases	Etiology	Medical treatment	Surgical treatment	Outcomes
Orbital infections	11	(i) Orbital reconstruction/Fracture repair 4/11 (36.4%) (ii) Orbital Implant 5/11 (45.5%)	Systemic ATB combination (>2 ATB) 6/11 (54.6%)	Excision of lesion 3/11 (37.5%)	(i) Resolution 7/10 (70%) (ii) VA of 20/200 or worse 3/10 (30%) (iii) Prolonged course 4/10 (40%)

Eyelid/periocular skin infections	28	(i) Ptosis Repair and/or Blepharoplasty 22/28 (78.6%) (ii) Implants 9/28 (32.1%)	Systemic ATB combination (>2 ATB) 14/28 (50%)	(i) Excision/incision and drainage/debridement 13/28 (46.4%) (ii) Removal of implant 7/28 (25%)	(i) Resolution 22/28 (78.5%) (ii) Prolonged course 6/28 (21.4%)

Lacrimal system infections	17	(i) Implants 13/17 (76.5%) (ii) Punctal Plug or Lacrimal Tube Insertions 9/17 (52.9%) (iii) DCR +/− stent placement 5/17 (29.4%)	Topical + systemic 7/17 (41.2%)	Removal of implant 10/17 (58.8%)	(i) Resolution 13/17 (76.5%) (ii) Prolonged course 3/17 (17.6%)

Keratitis	290	(i) LASIK 130/273 (47.6%) (ii) Trauma 43/264 (14.8%) (iii) Foreign body 51/211 (17.6%) (iv) Contact lenses 19/276 (6.4%) (v) Implants 44/254 (17.3%)	(i) Topical 108/203 (53.2%)^*^ (ii) Topical + systemic 85/203 (41.9%)^*^	(i) Removal of corneal flap 49/283 (17.3%) (ii) PKP 40/283 (14.1%)	(i) Resolution 190/235 (80.9%) (ii) VA 20/40 or better 112/204 (54.9%) (iii) VA of 20/200 or worse 40/204 (19.6%)

Scleritis	18	Scleral buckling 14/18 (77.8%)	Topical 16/17 (94.1%)	Explanation of buckle 13/17 (76.5%)	(i) Resolution 16/17 (94.1%) (ii) VA of 20/200 or worse 10/14 (71.4%)

Endophthalmitis	44	Cataract surgery (IOL insertion) 18/37 (48.6%)	(i) Intraocular + topical/systemic/periocular 14/38 (36.8%) (ii) Intraocular 5/38 (13.2%)	PPV 13/37 (35.1%)	(i) Resolution 12/36 (33.3%) (ii) Loss of eye 12/36 (33.3%) (iii) Loss of vision 12/36 (33.3%)

Uveitis	9	HIV/AIDS 5/9 (55.6%)	Systemic/topical + systemic 6/9 (66.7%)	(i) PPV 1/8 (12.5%) (ii) Evisceration 1/8 (12.5%) (iii) Enucleation 1/8 (12.5%)	(i) Resolution 4/9 (44.4%) (ii) Loss of eye 3/9 (33.3%) (iii) VA 20/40 or better 2/4 (50%)

Denominator differs from total number of cases due to missing data.

^*^Analysis was done based on infections that led to eventual resolution without loss of vision.

**Table 4:** Medical treatment of NTM keratitis.

Mode of delivery	Number of eyes *n* = 203
Topical	108 (53.2%)
Topical and systemic	85 (41.9%)
Systemic	5 (2.5%)
Topical, systemic, and periocular	2 (1%)
Topical and intraocular	1 (0.5%)
Topical and periocular	1 (0.5%)
Systemic and periocular	1 (0.5%)

Antibiotic (ATB) regimen	Number of eyes *n* = 192

Amikacin alone	56 (29.2%)
Amikacin + macrolide	27 (14.1%)
Amikacin + fluoroquinolone	24 (12.5%)
Amikacin + fluoroquinolone + macrolide	18 (9.4%)
Fluoroquinolone + macrolide	16 (8.3%)
Other^*^	14 (7.3%)
Fluoroquinolone alone	13 (6.8%)
Amikacin + 1 or more ATB^*^	11 (5.7%)
Macrolide + 1 or more ATB^*^	7 (3.6%)
Macrolide alone	5 (2.6%)
Fluoroquinolone + 1 more ATB^*^	1 (0.5%)

^*^Antibiotic not including amikacin/fluoroquinolone/macrolide.
